# Age-associated changes in the hippocampal-ventral striatum-ventral tegmental loop that impact learning, prediction, and context discrimination

**DOI:** 10.3389/fnagi.2012.00022

**Published:** 2012-08-08

**Authors:** Marsha R. Penner, Sheri J. Y. Mizumori

**Affiliations:** Laboratory of Neural Systems, Decision Science, Learning and Memory, Department of Psychology, University of WashingtonSeattle, WA, USA

**Keywords:** adaptive navigation, context discrimination, decision-making, error prediction, hippocampus, ventral striatum, ventral tegmental area

## Abstract

Studies of the neural mechanisms of navigation and context discrimination have generated a powerful heuristic for understanding how neural codes, circuits, and computations contribute to accurate behavior as animals traverse and learn about spatially extended environments. It is assumed that memories are updated as a result of spatial experience. The mechanism, however, for such a process is not clear. Here we suggest that one revealing approach to study this issue is to integrate our knowledge about limbic system mediated navigation and context discrimination with knowledge about how midbrain neural circuitry mediates decision-making. This perspective should lead to new and specific neural theories about how choices that we make during navigation determine what information is ultimately learned and remembered. This same circuitry may be involved when past experiences come to bias future spatial perceptions and response selection. With old age come not only important changes in limbic system operations, but also significant decline in the function of midbrain regions that underlie accurate and efficient decisions. Thus, suboptimal accuracy of spatial context-based decision-making may be, at least in part, responsible for the common observation of spatial memory decline in old age.

## Introduction

Cognitive map theory (O'Keefe and Nadel, 1978), which was based on the discovery of place cells in the hippocampus (O'Keefe and Dostrovsky, 1971), suggested that the hippocampus is important for the representation of experiences that occur within a spatial context (Nadel, 2008). These ideas have since been elaborated on, and one current assertion, discussed herein, is that the hippocampus does not just represent context, but also works to discriminate *between* contexts and to determine when a salient feature of a context have changed (Mizumori et al., 1999, 2007a). There is a growing body of experimental evidence providing support for this idea. Moreover, convergent evidence suggests a key role for the hippocampus in age-related impairments of context-based learning and memory. For example, work with rodent, primate, and human subjects shows that a compromised hippocampus results in spatial memory deficits that could be accounted for by poorer analysis of contextual details. These deficits are proposed to have a significant impact on context-dependent decision-making processes.

## What is context?

Although there may be some disagreement regarding what features make up or define a context, everyone would probably agree that a context is a multifaceted entity, and that everything we experience takes place within a context. According to Nadel (2008) the features that define a context must be relatively stable, meaning that their relationship to each other remains, even when there is no one there to experience them. Context, as we will use the term in the following discussion, refers to stable background stimuli, such as the geometrical features of a testing room, as well as more abstract features, such as the task demands and internal state that come to help make some aspects of a context more salient than others (see Smith and Mizumori, 2006a).

**Figure 1:**
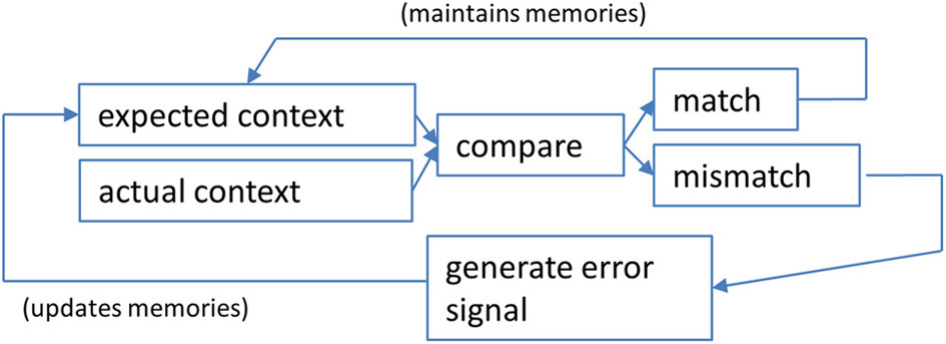
**A schematic illustration of the steps involved in a context prediction error analysis.** Information about expected features of a given context are compared against actual contextual features experienced by the animal. If they are perceived to be the same, a “match” signal is generated that maintains (or possibly strengthens) the neural network that underlies the current active memory. Pattern completion computations may predominate in such a determination of a match (see text for discussion). If a “mismatch” is detected, the result is hippocampal output that reflects the error in prediction. In this case, pattern separation computations may prevail over pattern completion computations. The impact of a context prediction error signal is to ultimately update long-term memories that will define the expected contextual features the next time an animal enters the same situation.

The detection of changes in context is necessary for both optimal learning and the selection of appropriate behaviors within a given context. In order to determine if a salient feature of a particular context has changed, match–mismatch comparisons are made (Figure 1). This allows an organism to determine how similar the current context is to the context the animal was expecting based on past experience (see Mizumori et al., 2007a).

### The hippocampus and context

There is growing evidence that the hippocampus has a special role in learning and memory because it functions to distinguish meaningful contexts, and in that way determine the saliency of different contexts (e.g., Mizumori et al., 1999; Smith and Mizumori, 2006a; Mizumori et al., 2007a; Penner and Mizumori, 2012). This function is critical for the formation of new episodic memories because it separates in time and space one meaningful event from the next. Such “chunking” of memories could facilitate long-term information storage according to memory schemas (Tse et al., 2007; Bethus et al., 2010).

Studies of the predominant type of neural representation by hippocampal pyramidal neurons, location-selective firing (O'Keefe and Dostrovsky, 1971; O'Keefe and Nadel, 1978), have provided insight into the mechanisms by which the hippocampus analyzes context information to result in context prediction error signals. As decades of research have shown (summarized in Mizumori et al., 2007a), “place fields” of recorded “place cells” are dynamic and integrated representations of multiple types of information, from sensory, motivational, and behavioral, to mnemonic. For example, changing any modality of cues, the motivational state, or the behaviors needed to perform the task result in alterations of place field properties, a process commonly referred to as re-mapping. Thus, it is the combination of these different types of information that have come to define the meaning of “context” when referring to hippocampal processing. The relative contributions of these different input types vary with task demands, and this is evidenced by findings across many laboratories that place fields change when rats use identical environmental information to solve tasks according to different strategies (e.g., Ferbinteanu and Shapiro, 2003; Kennedy and Shapiro, 2004; Mizumori et al., 2004; Eschenko and Mizumori, 2007). To the extent that different cognitive strategies are mediated by different underlying memories, a particular pattern of activated place cells is thought to reflect one memory, and a different pattern of activated place cells corresponds to a different memory (e.g., Samsonovich and McNaughton, 1997). Thus, when one refers to place field re-mapping, implicit is the notion that each map is driven by a different memory.

Many cleverly designed studies have sought to determine the features and sensory inputs that affect re-mapping of hippocampal place fields. Early studies, for example, showed that place cells are sensitive to changes in the visual environment (e.g., Ranck, 1973; O'Keefe, 1976; Olton et al., 1978; Muller and Kubie, 1987) such as the geometric features of the environment (e.g., Gothard et al., 1996; O'Keefe and Burgess, 1996; Wiener, 1996). Other sensory inputs can also affect place activity, including olfactory cues (Save et al., 2000), auditory cues (O'Keefe and Conway, 1978; McEchron and Disterhoft, 1999), and somatosensory cues (Young et al., 1994). Based on this body of work, it is clear that hippocampal pyramidal neurons process multimodal sensory cue information. Hippocampal place fields are also sensitive to changes in a task's reward structure (Smith and Mizumori, 2006b; Wikenheiser and Redish, 2011). Smith and Mizumori (2006b) tested this idea by training rats to distinguish Context A from Context B according to where reward was expected to be found. Importantly, the motivational, sensory and behavioral requirements of task performance were explicitly held constant across the two contexts so that changes in place fields could be attributed to the recall of a different memory. Place fields were found to reorganize at the beginning of trials in Context B, a time when a match–mismatch comparison may be implemented, and a time when there may be uncertainty about the context. In a similar experiment, Wikenheiser and Redish (2011) demonstrated that changes in reward contingency can modulate the trial-to-trial variability of hippocampal place cell activity.

## From prediction errors to context discrimination: a role for the hippocampus

What kind of computations take place in order for an organism to determine if a salient feature of the context has changed? When an organism's expectations about its experiences are violated, it is adaptive to update expectations so that adequate predictions can be made in the future. Once mismatches between expectations and outcomes no longer occur, learning is considered “complete.” According to classic learning theories, learning is driven by errors in the ability to accurately predict the occurrence of rewards, referred to as “prediction errors” (Rescorla and Wagner, 1972; Pearce and Hall, 1980; Sutton, 1988). The most common examples are the Rescorla–Wagner (Rescorla and Wagner, 1972) and temporal difference learning models (Sutton, 1988; Sutton and Barto, 1998) which use errors to drive associative changes. When a large prediction error is generated, a correspondingly large change in associative strength occurs. In contrast, no change in associative strength will occur if the computed prediction error is zero (i.e., the outcome is accurately predicted). Importantly, the relative sign of the error determines whether associative strength is promoted or weakened. When an outcome is better than predicted, the error generated will be positive, resulting in a strengthening of the association between the cues and the outcome. Alternatively, when the outcome is worse than predicted, a negative error signal is generated, and the association will weaken.

The neural instantiation of a reward prediction error was first observed in the midbrain dopamine system (Schultz et al., 1997). Using Pavlovian reward predicting paradigms in monkeys and rodents, it has been shown that dopamine cells increase phasic discharge when new rewards are encountered (Schultz et al., 1997). As the animal learns to expect reward, however, dopamine cell responses to rewards decline (Fiorillo et al., 2003). When this is the case, dopamine cells come to respond not to the reward itself but rather to the cues that predict the reward, such as a tone or light. The finding that reward responses of dopamine cells change from the reward to the cues that predict rewards is mirrored by corresponding changes in the timing of dopamine release in the nucleus accumbens (Day et al., 2007).

### Assessing value

**Figure 2:**
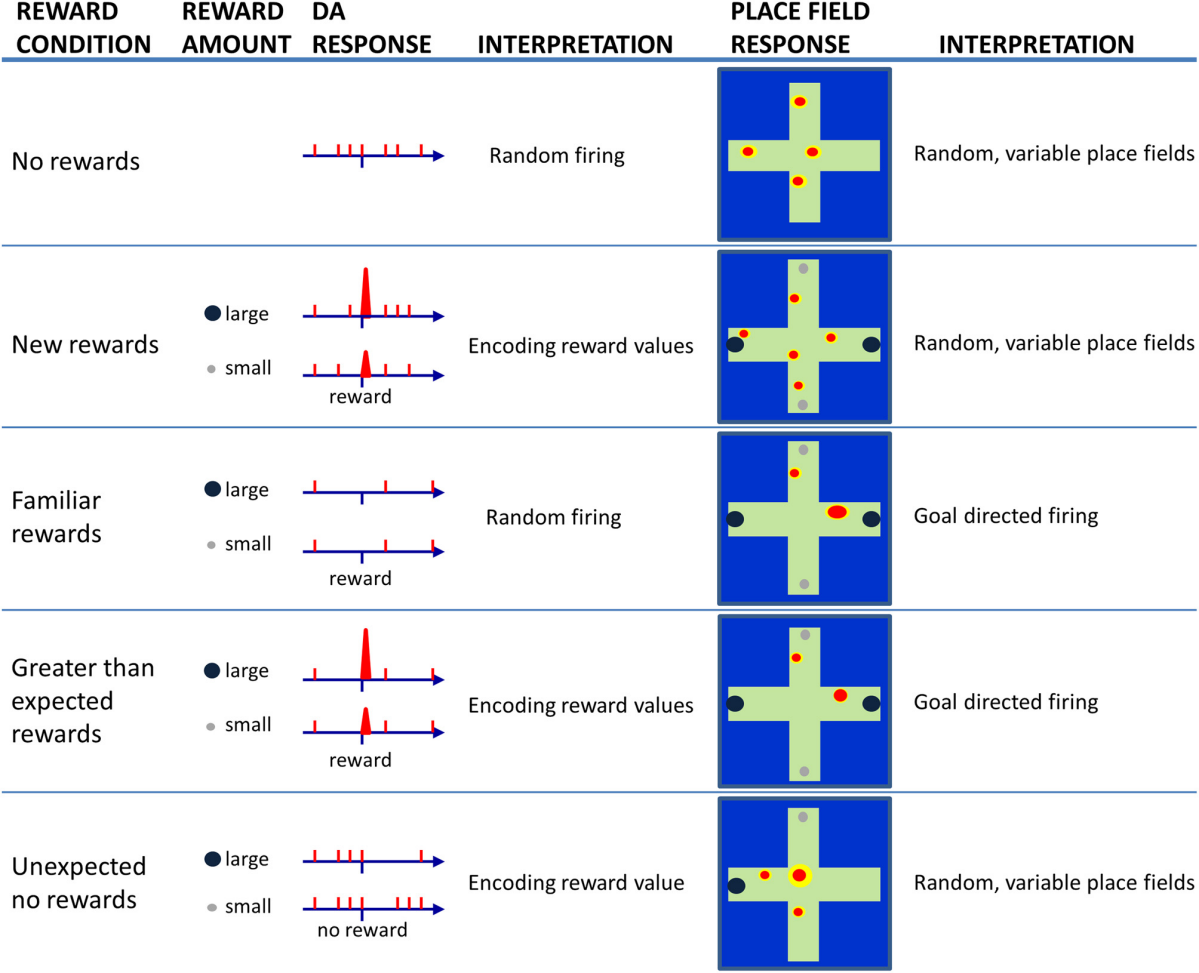
**Schematic illustration of dopamine (DA) cell responses to encounters of large or small rewards as a rat solves a maze task under different reward conditions (left).** Consistent with the literature, DA cells appear to fire randomly at low rates as animals traverse a maze when no rewards are present (No reward). When a rat encounters reward for the first time (New rewards), DA cells exhibit phasic burst firing to a larger extent following encounters with large rewards than small rewards. In this case, DA cells are considered to be encoding reward values. When the reward-finding task is learned (Familiar rewards), DA cells should no longer fire upon reward encounters. If the rat then unexpectedly encounters reward, one will again observe burst firing by DA cells in proportion to the magnitude of reward (Greater than expected rewards). If a trained rats goes to a location that was previously associated with rewards, but the reward is unexpectedly absent (Unexpected no rewards), then DA cells are observed to show brief periods of inhibited firing that is proportional to the amount of reward expected. The right panels illustrate expected (based on the current literature) place field responses when recorded under the same varying reward conditions. During random activity on a maze, place fields exist but they tend to occur in somewhat random locations. On a trial when new rewards are first encountered, place fields may continue to occur in random locations since the new reward information has yet to update long-term memories. As a task is learned, and locations are associated with specific rewards, one may observe increased place field specificity, and the location of the field may skew toward the rewarded location. If animals encounter larger than expected rewards, the place fields may not change or they may become even more specific to reflect the increased significance of rewarded locations. If on the other hand, a reward is not found at a previously rewarded location, then one may observe place fields to move, or re-map, due to the elevated degree of uncertainty.

The dopaminergic system is also part of a neural network that assesses the value of behavioral outcomes—reward-induced excitation of dopamine neurons scales to the magnitude of rewards (Schultz et al., 1997). Thus, encounters with large rewards are accompanied by larger amplitude phasic dopamine responses than encounters with small amounts of reward. In addition, dopamine cells respond to unexpected reward absences (Schultz et al., 1997) by decreasing their firing rates. The reduction in firing to the absence of expected reward is greater if the expectation was for a large, and not small, reward. A schematic illustration of these different dopamine cell responses to reward can be found in Figure 2 (left side of figure).

### Other functions of dopamine

In addition to generating a reward prediction signal, midbrain dopamine neurons also transmit signals related to salient but non-rewarding experiences such as aversive and alerting events (e.g., Pezze et al., 2001; Pezze and Feldon, 2004; Redgrave and Gurney, 2006). Thus, it has been suggested that dopamine neurons represent a heterogeneous population of cells that are connected to anatomically and functionally distinct brain networks (Bromberg-Martin et al., 2010). This would imply that dopamine neurons can have distinct roles in motivational control; some dopamine neurons may encode motivational value, supporting brain networks for seeking, evaluation, and value learning while others encode motivational salience, supporting brain networks for orienting, cognition, and general motivation (e.g., Berridge, 2007; Smith et al., 2011). For both types of dopamine neurons, an alerting signal could prime these cells for rapid detection of potentially important cues, a computation that would be necessary for detecting salient aspects and/or changes in context (see Bromberg-Martin et al., 2010).

### Prediction errors in other brain regions

Neural correlates of reward prediction errors have also been observed outside of the midbrain, in the prefrontal cortex, orbitofrontal cortex, striatum, amygdala, reticular formation, and habenula (Nobre et al., 1999; Schultz and Dickinson, 2000; McClure et al., 2003; Satoh et al., 2003; Bayer and Glimcher, 2005; Fiorillo et al., 2005; Tobler et al., 2006; Yacubian et al., 2006; Belova et al., 2007; Matsumoto and Hikosaka, 2007; Roesch et al., 2007; Puryear and Mizumori, 2008). This suggests that a critical and common function of many brain areas is to provide an outcome expectancy signal or prediction. Evidence is accumulating that the hippocampus is another brain region that may generate error prediction signals.

### Hippocampal context prediction error

It has been suggested that hippocampal neurons represent the contextual features of an environment for the purpose of computing the extent to which familiar contexts change (Nadel and Wilner, 1980; Nadel and Payne, 2002), or more specifically the extent to which expected and actual context features match (e.g., Mizumori et al., 1999; Anderson and Jeffery, 2003; Jeffery et al., 2004; Hasselmo, 2005; Smith and Mizumori, 2006a,b; Nadel, 2008). If the hippocampus determines that there was no change (e.g., a familiar context appears as expected), then the currently active neural network that defines the current memory will be strengthened so that it can continue to drive behavior. If, on the other hand, a mismatch signal is generated, it will alert other neural systems of the brain so that they become prepared for rapid and new learning (Figure 1). The mismatch could be considered an example of an error in predicting the contextual details of the current situation and is thus referred to here as a “context prediction error.” Transmission of a context prediction error signal can inform distal brain areas that a change in the context has occurred. For example, upon receipt of the hippocampal message, midbrain structures may respond with changes in excitation or inhibition to determine the subjective value of the context prediction error signal. Similarly, the same hippocampal signal may enable plasticity mechanisms (perhaps in neocortex) that allow new information to be incorporated into existing memory schemas (e.g., Mizumori et al., 2007a,b; Tse et al., 2007; Bethus et al., 2010). In this way, hippocampal context analyses becomes critical for the formation of new episodic memories via a prediction signal that can provide a mechanism that separates in time and space one meaningful event from the next. Figure 2 shows schematized hippocampal place field responses, based on the vast literature on place field responses to context changes (Mizumori et al., 2007b), that illustrate the relationship between hippocampus and dopamine neurons: when the hippocampus signals a change in context, dopamine cells encode reward value.

## Hippocampal changes in the aged brain

The natural environment in which we behave continually changes. Optimal learning and performance depends on our ability to detect changes in context with sufficient temporal and informational specificity so that decisions about future adaptive responses can be made. Alterations in the ability of the hippocampus to perform these operations will contribute to significant learning and memory deficits that are manifested by an inability to recognize that something important or salient has changed, as is often observed in aged subjects. There is abundant and consistent evidence from aged rodents, monkeys, and humans that behavioral deficits are often observed on tasks whose optimal solution requires the use of spatial information (Gallagher and Rapp, 1997; Rosenzweig and Barnes, 2003; Wilson et al., 2006). Although neurobiological differences certainly exist across species, the organizational and functional principles governing spatial information processing are strikingly similar from rat to human.

The hippocampal place cells of aged rats show changes that are likely to significantly impact memory and context analysis functions. The initial pioneering work of Barnes (1979) demonstrated significant age-related deficits in spatial memory function that correlated with changes in synaptic plasticity; modification of synaptic efficacy at the perforant path synapses in the dentate gyrus was found to be significantly impaired (Barnes, 1979). Subsequent to this work, Barnes et al. (1983) recorded CA1 place cell activity in adult and aged rats as they performed a forced-choice eight-arm maze task. Rats were not required to remember any spatial information but could access reward arms sequentially. Place fields in aged rats were found to be less place-specific and less reliable, in that they did not fire every time an animal ran through the field. These changes were suggested to contribute to the memory deficits observed in aged rats (Barnes et al., 1983).

Subsequent to this work, Barnes et al. (1997) demonstrated that when aged rats are brought into a familiar environment, they occasionally re-map. That is, there was an occasional global change in the place fields of most or all of the cells being recorded—some fields changed their preferred location within the environment, some cells stop firing in a previously preferred location (i.e., lose a place field), and some previously silent cells began firing (i.e., gained a place field). When this kind of re-mapping occurred during a recording session, the overall place fields appeared “noisy,” as was initially demonstrated by Barnes (Barnes et al., 1983). Importantly, when this re-mapping occurred it did so only when animals re-entered an environment it had previous experience with, never when an animal remained in the environment (Barnes et al., 1997).

Subsequent work, however, reported conflicting results in that the place fields of aged rats were shown to be just as place-specific and stable as those of adult rats, and under some conditions, more so (Markus et al., 1994; Mizumori et al., 1996; Shen et al., 1997; Tanila et al., 1997; Oler and Markus, 2000; Wilson et al., 2003). For example, Tanila et al. (1997) manipulated visual cues within the recording environment, and found that aged, memory-impaired rats were less likely than were adult rats or aged, memory intact rats to re-map in response to major changes in the environment. Thus, instead of being multi-stable, as reported by Barnes et al. (1997), the place cells of aged memory-impaired rats were often impervious to changes in the visual environment.

These results were subsequently reconciled by Wilson et al. (2004) who recorded place cells from aged and young rats as they repeatedly explored either a highly familiar environment or a novel environment. Initially, place cells in aged rats maintained their activity between the familiar and novel environments, suggesting that they were more rigid than those of the younger rats, as had been previously reported by others. However, Wilson et al. (2004) also observed that the rigidity of aged place cells was temporary. With additional experience, new representations of the novel environment could eventually be formed by aged rats, indicating a delay rather than an inability to detect a change in the environment. Finally, once these new spatial representations did develop, they were shown to be multi-stable across repeated exposures to the formerly novel environment. Thus, it appears that significant deficits in the ability of visual cues to control spatial representations of aged rats can manifests in at least three ways: rigidity, delayed control by external cues, and multi-stability (Wilson et al., 2004).

Environments or contexts are composed of more than just visual cues, and these other features can also exert control over place cell firing. Oler and Markus (2000) tested the idea that task demands, rather than spatial cue-based manipulations, would differentially impact changes in place field characteristics in young and aged rats. To test this idea, rats moved through a figure eight maze to retrieve chocolate rewards. The maze configuration was then changed to a plus maze configuration. Importantly, the maze remained in the same testing environment, so visual cues within the testing room remained unchanged. Rats were then required to run the plus maze, and after several laps, the maze was again restored to a figure eight configuration. Recordings from hippocampal place fields changed their spatial firing preference between the two maze configurations in younger rats, but this change did not occur in aged rats. Instead, place cells in the aged hippocampus retained their spatial firing pattern even though the task demands had changed. These results demonstrate that hippocampal place cells in young rats encode task-related information within a relatively stable spatial context while aged rats show deficits in encoding this information. The possibility remains that old rats were simply delayed in encoding changes in task demands, as described by Wilson et al. (2004). This idea has not yet been explicitly tested.

The work discussed thus far pooled data obtained from different subregions of the hippocampus (CA1 and CA3) in order to increase statistical power. It is now appreciated, however, that subareas of the hippocampus perform different information processing tasks and have different place field characteristics. These differences are a direct result of differences in their efferent and afferent projections, and this makes them differentially sensitive to context manipulations that produce re-mapping. For example, the CA3 subregion receives excitatory inputs from the mossy fibers of dentate granule cells, layer II of the entorhinal cortex, as well as a recurrent network of densely interconnected CA3 pyramidal cells, making it particularly suited for rapid learning of environment-specific features (Nakazawa et al., 2003). Work by several groups have shown that CA3 pyramidal cells exhibit an all-or-nothing change in their firing patterns when familiar environments are changed, or new environments are encountered (Lee et al., 2004; Leutgeb et al., 2004; Vazdarjanova and Guzowski, 2004; Miyashita et al., 2009). Conversely, area CA1 receives excitatory input from CA3 via Schaffer collaterals and the entorhinal cortex but has extremely limited intrinsic excitatory connections. When changes to familiar environments are encountered, area CA1 has been shown to display a gradual change in firing patterns. This has led some to suggest that area CA1 acts as a “comparator,” in that it determines if a change in context has occurred by comparing the outputs of CA3 with direct inputs from the entorhinal cortex (Mizumori et al., 1999; Leutgeb et al., 2004; Vazdarjanova and Guzowski, 2004).

To test the idea that specific subregions of the hippocampus may be selectively vulnerable to effects of the normal aging processes, Wilson et al. (2004) compared the spatial firing patterns of CA1 and CA3 neurons in aged memory-impaired rats with those of young rats as they explored familiar and novel environments. Within area CA1, place cells in aged and young rats had similar firing characteristics in both familiar and novel environments. In contrast, within area CA3, aged rats showed place cells with higher firing rates when compared to young rats. In addition, CA3 place cells of aged rats failed to change their firing rates and place fields to the same degree that CA3 cells of young rats did when the rats were introduced to a novel environment. Thus, aged CA3 cells failed to rapidly encode new spatial information compared with young CA3 cells, suggesting a unique contribution of CA3 dysfunction to age-related memory impairment and context discrimination. In fact, these results are supported by the finding that a complete ensemble of CA3 pyramidal neurons are activated by a single exposure to a context, whereas CA1 cells require multiple exposures in order for a complete ensemble to be activated (Miyashita et al., 2009).

The dentate gyrus is another subregion of the hippocampus that is likely to contribute to a context discrimination function. Because sparse firing patterns are characteristic of dentate granule cells make it technically challenging to record from these cells, a relative dearth of *in vivo* electrophysiology data has been collected from this brain region. Other methods, including immediate-early gene imaging and fMRI studies have provided extensive evidence that this subarea of the hippocampus is particularly vulnerable to normative aging processes (Small et al., 2004; Penner et al., 2011).

## Computational mechanisms that support context discriminiation

Detecting a change in context is a necessary computation if one needs to discriminate contexts. Originating in the work of Marr (1971), pattern separation and completion computations are two functions that are widely attributed to specific subregions of the hippocampus (Marr, 1971; McNaughton and Morris, 1987; O'Reilly and McClelland, 1994; Samsonovich and McNaughton, 1997). Pattern completion is the process through which incomplete, noisy or degraded input are filled in based on representations that have previously been stored. In this way, complete episodes/memories can be recalled without a complete set of inputs. Pattern separation, on the other hand, is a computation that orthogonalizes similar inputs/representations. This functions to make similar inputs as dissimilar as possible, so that similar episodes/memories can be distinguished (Guzowski et al., 2004; Yassa and Stark, 2011). The interplay between these processes determines the extent to which hippocampal output signals a context prediction error: if pattern completion is greater than pattern separation, the hippocampal output signals a “match,” whereas conditions in which pattern separation is greater than pattern completion should generate a mismatch (or prediction error) signal (Mizumori, 2008). Specific subareas of the hippocampus may compute the degrees of pattern separation and completion. Both theoretical models and experimental work suggest that the granule cells of the dentate gyrus perform pattern separation functions on the input received from the entorhinal cortex. Area CA3 is envisaged as an auto associative network and is therefore ideal for pattern completion functions on inputs received from the dentate gyrus and its own recurrent collaterals. Because of the unique pattern of excitatory inputs to area CA3, this subarea can also perform a pattern separation function. This function may be supported by a subset of neurons within the entorhinal cortex that bypass the dentate gyrus, and thus provide direct input to area CA3. This weaker input can compete with the input from mossy fibers of the dentate gyrus, and thus area CA3 may be well equipped to perform both pattern completion and pattern separation functions (Guzowski et al., 2004; Yassa and Stark, 2011).

In sum, the result of pattern separation and pattern completion computations determines the extent to which hippocampus generates a context prediction signal that can be broadcast to other brain regions for the purposes of modifying behavior that is appropriate for the current context. The work discussed here suggests that aged rats show impairments in both pattern separation and pattern completion computations, resulting in hippocampal representations of contexts that can be either too rigid, or too plastic. Impaired pattern completion in aged rats might cause the occasional retrieval of an incorrect map (especially for CA3 place fields) upon entry to a familiar environment, while impaired pattern separation in aged rats might prevent the formation of a new map in response to environmental changes (Yassa and Stark, 2011).

As discussed above, studies of place cell firing characteristics between adult and aged rats initially produced conflicting results. In some cases, it appeared that hippocampal cells engaged in too much pattern separation, resulting in multi-stable or unstable place fields, while in other instances, it appeared that the reverse was true (e.g., Barnes et al., 1997; Tanila et al., 1997). These results were subsequently reconciled (Wilson et al., 2004, 2005), especially in the case when recordings from CA3 and CA1 were analyzed separately (Wilson et al., 2005). These processes have since been investigated in human subjects (e.g., Bakker et al., 2008; Lacy et al., 2011), and recent work has shown age-related changes in these processes (Stark et al., 2010; Yassa et al., 2011a,b; Holden et al., 2012). For example, Yassa et al. (2011a) tested the ability of young and aged adults to discriminate between novel, familiar or similar objects and with measuring BOLD activity in the hippocampus during performance of the task. They observed a behavioral impairment in pattern separation for the aged adults compared to the young controls. These findings were related to an increase in CA3/dentate gyrus activity, a finding reminiscent of animal work showing hyperactivity in area CA3 (Wilson et al., 2004). In addition, Yassa et al. (2011a) found that larger changes in the input (greater dissimilarity) were necessary in order for aged adults to successfully encode new information as distinct from previously learned information.

## Targets of the context prediction signal

The hippocampus is hypothesized to provide a context prediction error signal to other areas of the brain in order to determine if expectations about the current context have been met, or if they need to be updated. The hippocampus is directly connected to both the ventral striatum (also known as the nucleus accumbens) and the prefrontal cortex, and these structures are therefore direct recipients of the hippocampal context prediction error. In addition, the hippocampus regulates activity within the ventral tegmental area (VTA) via its effects on the ventral striatum (Yang and Mogenson, 1987; Floresco et al., 2001) and via the lateral septum (Luo et al., 2011). The following discussion will focus on contextual information processing within the hippocampal-ventral striatal-VTA loop (Voorn et al., 2004; Pennartz et al., 2009, 2011; Humphries and Prescott, 2010).

### Hippocampus-ventral striatum interactions

The ventral striatum is often referred to a limbic-motor interface (Mogenson et al., 1980). It receives convergent glutamatergic input from multiple sensory and association areas of the neocortex, and the limbic system, including the hippocampus and related structures (for review see Humphries and Prescott, 2010). The nucleus accumbens, the main structure of the ventral striatum, can be divided into core and shell subregions, which differ significantly in terms of their cellular morphology, neurochemistry, and patterns of projections. The shell receives hippocampal input predominantly from ventral CA1 and subiculum, whereas the core receives it from dorsal CA1 and subiculum and from parahippocampal regions (Voorn et al., 2004).

The ventral striatum is not only important for processing spatial and contextual cues (Annett et al., 1989; Seamans and Phillips, 1994; Floresco et al., 1997; Ferretti et al., 2010), but also processes information relevant to effort and cost-based decision-making (e.g., Aberman et al., 1998; Aberman and Salamone, 1999; Hauber and Sommer, 2009; Day et al., 2011). The ability to make these kinds of decisions is essential if animals are to make adaptive behavioral choices within a given context. The ventral striatum appears strategically positioned to play a key role in context-dependent value-based decisions given the convergent evidence from a variety of maze studies, including the spatial version of the Morris swim task (Setlow and McGaugh, 1998; Sargolini et al., 2003), the radial maze (Gal et al., 1997; Smith-Roe et al., 1999), a spatial version of the hole board task (Maldonado-Irizarry and Kelley, 1995), as well as a task in which the animals are required to discriminate a spatial displacement of objects (Annett et al., 1989; Seamans and Phillips, 1994; Ferretti et al., 2010).

To investigate the idea that the ventral striatum associates spatial context with reward information, Lavoie and Mizumori (1994) recorded neural activity in the ventral striatum while rats navigated an eight-arm radial maze for food reward. This study demonstrated, for the first time, spatial firing correlates within the ventral striatum. The mean place specificity for all ventral striatal neurons was significantly lower than that typically observed in the hippocampus (Barnes et al., 1990), indicating that while ventral striatal neurons discharge with spatial selectivity, they are not as selective as those observed from hippocampal neurons. The moderate spatial selectivity likely reflects the integration of spatial with other non-spatial information within the ventral striatum, including reward and movement. The fact that single ventral striatal neurons encode multiple types of information supports the view that spatial, reward, and movement information may be integrated at the level of individual ventral striatal neurons (Lavoie and Mizumori, 1994; Pennartz et al., 2011). Recent evidence suggests that spatial information within the ventral striatum is derived from the hippocampus. Ito et al. (2008) showed that an interruption of information sharing between the hippocampus and shell of the nucleus accumbens disrupted the acquisition of context-dependent retrieval of cue information, suggesting that the shell, in particular, may provide a site at which spatial and salient cue information may be integrated.

### From the ventral striatum to midbrain

**Figure 3:**
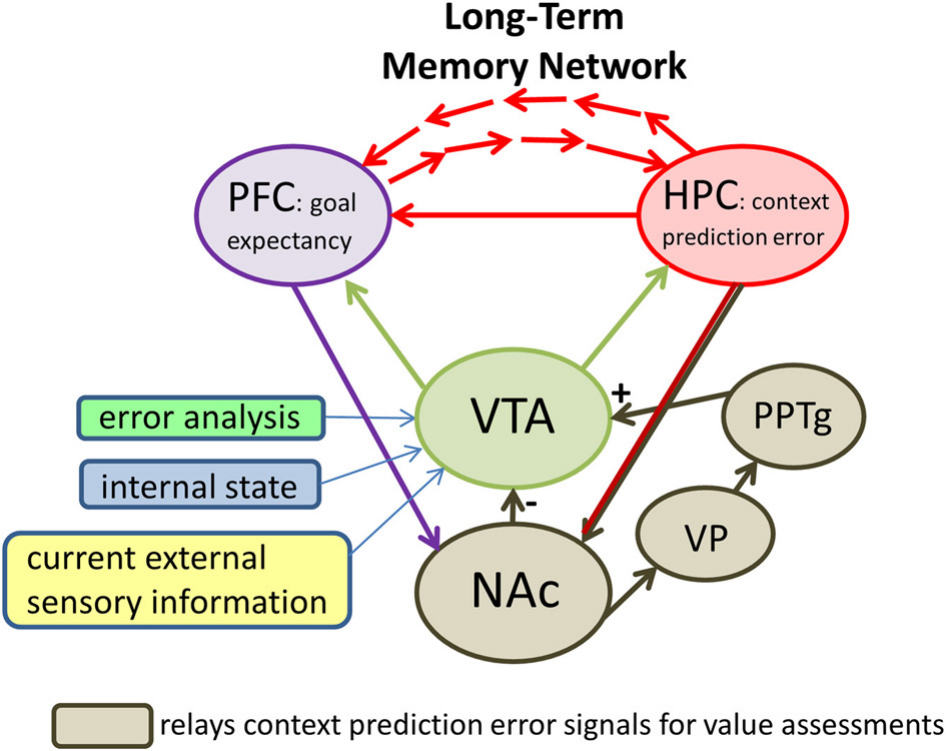
**A proposed neural circuit illustrating that midbrain (dopamine) systems and the prefrontal cortex (PFC) are key brain areas that evaluate the significance of hippocampal (HPC) context prediction error signals for the purpose of directing future behaviors and updating long-term memories.** Direct HPC output arrives in the midbrain system via projections to the ventral striatum (i.e., the nucleus accumbens, or NAc). The NAc determines whether the outcomes of behavior are as predicted based on an animal's expectations for a given context. If the outcome is as expected, NAc continues exerting inhibitory control (−) over ventral tegmental area (VTA) neurons. In this case, encounters with rewards do not result in dopamine cell firing. In constrast, context prediction error signals from the HPC to the NAc may preferentially excite (+) VTA neurons via an indirect pathway that includes the ventral pallidum (VP) and the pedunculopontine nucleus (PPTg). The result of this elevated excitation may be a depolarization of VTA neurons such that they are more likely to fire when subsequent reward information arrives in VTA. Also influencing the likelihood that dopamine cells will fire in response to future rewards are specific computations that reflect negative reward prediction errors (reward prediction error analysis), information about the animal's current motivational and behavioral state (internal state), and additional information about the current salient cues (current external sensory information). The latter appears to involve at least the PPTg. The impact of the prefrontal cortex on VTA function is not clear. One possibility is that it provides the midbrain circuitry with information about what to expect in terms of goals based on past experience. The summed input to the VTA results in an output that reflects the subjective value of context prediction error signals.

Signals originating in the nucleus accumbens impact VTA dopamine neurons via two main routes (Figure 3): a direct inhibitory GABAergic synaptic input from the accumbens itself (e.g., Heimer et al., 1991; Kalivas et al., 1993) or an indirect route that includes the ventral pallidum and the pedunculopontine nucleus (PPTg; e.g., Floresco et al., 2003; Zweifel et al., 2009). The direct route may relay information about the expected features of a context while the indirect route may relay information about the actual context features (Humphries and Prescott, 2010; Penner and Mizumori, 2012). Since the direct projection is inhibitory onto dopamine cells, and the indirect route is excitatory, hippocampal signals indicating that a familiar context has not changed (i.e., there is no mismatch of expected and actual context information) would result in equal input from direct (−) and indirect (+) pathways. The result is that dopamine cells should show no change in baseline responding when rewards are encountered. Indeed, under such circumstances dopamine cells do not respond to rewards (e.g., Schultz et al., 1997). In the event that hippocampus signals a change in context (i.e., a mismatch between expected and actual contextual features) the indirect input would be stronger than the direct input. The resultant increased excitability after input from the indirect pathway could make more likely a dopamine cell response to a subsequent reward encountered. Indeed dopamine cells are known to increase responding to rewards after a context change (e.g., Schultz et al., 1997; Puryear et al., 2010). Thus dopamine neurons may be placed on high alert after a change in context is detected, and in that way be better prepared to determine the subjective value of the hippocampal prediction error signal.

### Aging of the ventral striatum

There is a limited literature investigating the effects that the normal aging process has on function of the ventral striatum. Much of what is known has come from human work. For example, Schott et al. (2007) used fMRI to investigate the neural mechanisms underlying reward prediction and reward outcome processing in young and elderly healthy subjects. Young adults showed a pattern of midbrain and ventral striatal activation for cues that predicted monetary reward when compared with cues that predicted neutral feedback. In contrast, healthy aged subjects showed the opposite pattern: an absent reward prediction response in the face of mesolimbic activation to reward feedback itself. This may reflect a reduced ability of older subjects to accurately estimate expected rewards. These results support other behavioral results indicating that older adults have deficits in learning from positive feedback (e.g., Mell et al., 2005).

### Hippocampal-VTA interactions

The VTA and hippocampus reciprocally interact such that novel, context information detected by the hippocampus enhances VTA dopamine release. This in turn enables encoding of new information into long-term memory (Lisman and Grace, 2005; Bethus et al., 2010). Input from the VTA to the hippocampus is direct (Gasbarri et al., 1994), whereas hippocampal output to VTA is indirect, arriving via the lateral septum (Luo et al., 2011) and the ventral striatum (Yang and Mogenson, 1987; Floresco et al., 2001). As evidence of the functional significance of VTA-hippocampus interactions, the VTA has been shown to regulate hippocampal activity and spatial learning (Martig et al., 2009; Martig and Mizumori, 2011a,b) and encoding of hippocampus-dependent memories (Rossato et al., 2009). In addition, the hippocampus has been shown to regulate dopamine responses to novelty (Legault and Wise, 2001) and analogous to hippocampal place fields, phasic reward responses of putative dopamine neurons in VTA are sensitive to changes in the visuo-spatial context (Puryear et al., 2010).

Recent work by Luo et al. (2011) has identified a circuit from area CA3 of the dorsal hippocampus to the VTA that uses the lateral septum as a relay. When area CA3 is stimulated, dopaminergic neurons within the VTA are excited, while non-DA neurons are inhibited. The observed excitation of dopamine neurons is likely mediated by disinhibition because local antagonism of GABA receptors can block the response to CA3 stimulation. Conversely, inactivating components of this circuit blocked evoked responses in VTA and also had a significant impact on reinstatement of drug-seeking by contextual stimuli. Thus, the link between the hippocampus and the VTA may be an important substrate by which information about the environmental context regulates goal-directed behavior. Efficient reward-seeking requires that environmental stimuli be interpreted, allowing accurate predictions about when and where reward can be expected. It is possible that dorsal CA3 conveys information to VTA about the current context as a whole, which allows rapid activation of dopamine neurons to promote salience attribution to conditioned contexts. Such processing is important for cognitive function by providing adjustments in behavior in response to changing real-world environments.

### Aging of the dopamine system

A number of age-related changes in the dopamine system have been demonstrated (for review, see Backman et al., 2006, 2010). Most of this work has been done in non-human primate and human subjects and have for the most part focused on changes that occur in the striatal and frontal cortical areas of the brain. For example, dopamine concentration (Goldman-Rakic and Brown, 1981), transporter availability and binding potential (e.g., Volkow et al., 1998a,b; Mozley et al., 2001), and dopamine D1 and D2 receptor density (e.g., Volkow et al., 1996; Wang et al., 1998; Backman et al., 2000) all decline with age. In addition, work by Kaasinen et al. (2000) found significant age-related declines of D2/3 receptors in all brain regions studied, including the frontal cortical areas, and the hippocampus (Kaasinen et al., 2000). Importantly, for all of the studies mentioned here, these changes occur in the absence of pathological aging, such as Parkinson's and Alzheimer's disease. Treatment of aged monkeys with a D2 receptor agonist reduces the decline in performance on a delayed memory task (Arnsten and Goldman-Rakic, 1985). In addition, imaging of D2 receptors in humans has found a correlation between receptor availability and performance on attention and response inhibition tasks and on the Wisconsin Card Sorting Task, and have also shown that striatal D2 receptor binding accounts for a greater amount of variation in performance on processing speed and episodic memory tasks than does chronological age (Volkow et al., 1998a; Backman et al., 2000). Based on these findings, it has been proposed that age-related deficits in learning are the result of a decline in dopaminergic function in older age (Nieuwenhuis et al., 2002; Backman et al., 2006, 2010). To date, data on the effects that the normal aging process may have on the midbrain, especially the VTA itself are sparse. However, there is some evidence indicating that significant age-associated changes in dopamine transporter activity within the VTA (e.g., Cruz-Muros et al., 2009).

## The impact of aging on context-dependent decision-making behaviors

Decision-making is the process of choosing an option or course of action from among a set of alternatives. This process depends on the decision-maker's estimate of the outcome of the different options (Rangel et al., 2008). Because memory has a significant impact on the ability of an organism to make the “best” decision in a given context, it may not be surprising that aging affects decision-making behaviors. Determining the subjective value of behavioral outcomes requires an assessment of the extent to which expected rewards are actually received. While it is true that a number of factors are likely to contribute to the definition of one's expectations for behavioral outcomes, the most obvious factor is whether there is an expectation based on past experience. Presumably if there is no history of obtaining rewards in a particular context, then there should be no expectation for a reward. If, however, there is some kind of expectation of reward because of past experiences, then the degree of expectation (i.e., subjective goal values) can be enhanced or reduced (Schultz et al., 2008). The ability to learn from feedback and adaptively change behavior according to positive and negative outcomes is significantly impaired in aged subjects (Samanez-Larkin et al., 2007; Zamarian et al., 2008; Eppinger et al., 2010, 2011; Herbert et al., 2011). In addition, older adults are impaired at learning if reward delivery is probabilistic, a time when predicted outcomes are unreliable. When, however, reward contingencies are deterministic (certain) older adults are able to learn as well as young adults. Older adults' learning impairments under reward uncertainty may reflect deficits in the ability to form and update value (outcome) representations using prediction error signals. Since learning and the formation of new memories is driven by the ability to respond to prediction error signals, it would be expected that individuals (e.g., aged adults) who suffer from responding appropriately to error signals would also show impaired learning and memory.

Assessments of subjective goal values, then, may change when hippocampus generates context error signals. The ultimate dopamine cell response to such hippocampal input reflects a subjective value that can be ascribed to the context change. How subjective value is computed is not understood. One factor that is sure to come into play is efficient learning strategies within a given context. For example, if an aged organism is unable to learn about the reward contingencies within a given environment, perhaps because of insufficient or erroneous prediction codes, then adaptive choices within a given context will be impaired. It appears that regulation of the dopamine cell phasic firing by hippocampus may control the “teaching” signal that is often attributed to dopamine neurons (e.g., Luo et al., 2011), a signal that informs neural circuitry that makes decisions about the selection of future responses.

## Summary and suggestions for future work

The work presented here highlights a fundamental role for the hippocampus in context discrimination. While the hippocampus may be especially important for detecting and responding to the spatial features of context, it is becoming increasingly clear that the hippocampus is also sensitive to non-spatial aspects of a context, including other sensory cues, task demands, and internal factors such as hunger or thirst. When salient aspects of a context change, the hippocampus is in a unique position to broadcast a teaching signal in the form of a context prediction error signal to other brain areas to which it is closely connected, including the ventral striatum and VTA. These brain regions then assist the hippocampus in determining if salient features of a context have, in fact, changed. Functional changes in the hippocampus that occur during the normal aging process result in significant deficits in the ability to accurately determine if a change in context has occurred. For example, an age-related deficit in the ability to discriminate contexts by the hippocampus will result in inefficient signals that would normally prepare other connected brains regions (e.g., the midbrain, ventral striatum, and prefrontal cortex) for the plasticity required to adaptively respond. Although not discussed here, additional brain regions are likely to contribute to the learning and memory deficits observed in aged subjects. For example, the prefrontal cortex, dorsal striatum, and amygdala are likely to contribute to context discrimination processes under certain conditions.

Dopamine signaling within the VTA has a significant impact on the ability of both the ventral striatum and the hippocampus to integrate reward and spatial information. Changes in dopaminergic signaling, then, are also important for determining if salient changes in context have occurred. Although not discussed, there is experimental evidence that other neuromodulatory systems, including acetylcholine and serotonin are affected by normative aging processes (see Wilson et al., 2006; Eppinger et al., 2011) and may contribute to a failure to accurately detect contextual changes. In addition, although it is clear that many changes in dopamine signaling occur, evidence remains relatively sparse in terms of how age affects information processing within the VTA itself. Thus, this is a particular area of research ripe for further investigation.

### Conflict of interest statement

The authors declare that the research was conducted in the absence of any commercial or financial relationships that could be construed as a potential conflict of interest.
